# Genetic etiological analysis of auditory neuropathy spectrum disorder by next-generation sequencing

**DOI:** 10.3389/fneur.2022.1026695

**Published:** 2022-12-08

**Authors:** Lianhua Sun, Zhengyu Lin, Jifang Zhang, Jiali Shen, Xiaowen Wang, Jun Yang

**Affiliations:** ^1^Department of Otorhinolaryngology-Head and Neck Surgery, Xinhua Hospital, Shanghai Jiao Tong University School of Medicine, Shanghai, China; ^2^Shanghai Jiao Tong University School of Medicine Ear Institute, Shanghai, China; ^3^Shanghai Key Laboratory of Translational Medicine on Ear and Nose Diseases, Shanghai, China

**Keywords:** auditory neuropathy spectrum disorder, targeted next-generation sequencing, gene mutation, etiological analysis, minigene assay

## Abstract

**Objective:**

Auditory neuropathy spectrum disease (ANSD) is caused by both environmental and genetic causes and is defined by a failure in peripheral auditory neural transmission but normal outer hair cells function. To date, 13 genes identified as potentially causing ANSD have been documented. To study the etiology of ANSD, we collected 9 probands with ANSD diagnosed in the clinic and performed targeted next-generation sequencing.

**Methods:**

Nine probands have been identified as ANSD based on the results of the ABR tests and DPOAE/CMs. Genomic DNA extracted from their peripheral blood was examined by next-generation sequencing (NGS) for a gene panel to identify any potential causal variations. For candidate pathogenic genes, we performed co-segregation among all family members of the pedigrees. Subsequently, using a mini-gene assay, we examined the function of a novel splice site mutant of *OTOF*.

**Results:**

We analyzed nine cases of patients with ANSD with normal CMs/DPOAE and abnormal ABR, discovered three novel mutants of the *OTOF* gene that are known to cause ANSD, and six cases of other gene mutations including *TBC1D24, LARS2, TIMM8A, MITF*, and *WFS1*.

**Conclusion:**

Our results extend the mutation spectrum of the *OTOF* gene and indicate that the genetic etiology of ANSD may be related to gene mutations of *TBC1D24, LARS2, TIMM8A, MITF*, and *WFS1*.

## Introduction

Afferent nerve conduction problems combined with the proper operation of outer hair cells enduring otoacoustic emissions (OAE) and/or cochlear microphonics (CMs) are the hallmark symptoms of auditory neuropathy spectrum disease (ANSD) (1). The first case of ANSD was diagnosed by Starr et al. (2), who found that 10 individuals had abnormal peripheral auditory neural transmission but normal outer hair cell function. The incidence of ANSD, on the other hand, is not fully clear, with studies reporting incidences ranging from <1% to almost 10% in patients with hearing impairment (3–6). The wide range of clinical characteristics in patients with ANSD in different studies (3) is reflected in the large variety of prevalence. Meanwhile, a variety of etiologies have been identified, including genetics, dysmaturity, cochlear nerve abnormalities, and prenatal infections such as measles, mumps, or cytomegalovirus—CMV. Prematurity, prenatal conditions, such as severe icterus and kernicterus, hypoxia induced by mechanical ventilation, septicemia, ototoxic medications, and meningitis were listed as postnatal causes, which might manifest symptoms later in life (3, 7, 8). Cochlear implantation is thought to be the best therapeutic option for patients with ANSD. Nevertheless, due to the widely diverse clinical treatment results, patients with ANSD who accepted cochlear implantation may experience faulty language and speech outcomes. Pre- or post-synaptic lesions or disorders of the central nervous system with hypoplasia of the auditory nerve may be relevant in this regard (9–15).

A total of 13 genes have been identified as causing ANSD thus far (16). Mutations of *OTOF, PJVK*, and *DIAPH3* are the most common hereditary causes of isolated ANSD (17), while *OTOF* mutations account for more than 18–41% of congenital individuals with ANSD in China (18, 19). To date, over 110 *OTOF* mutations have been identified (Human Gene Mutation Database).

In this study, we screened 9 cases of patients with ANSD who had normal results in distortion product otoacoustic emissions (DPOAE)/cochlear microphonic potentials (CM) but abnormal auditory brainstem responses (ABR); of which, we discovered 3 cases of *OTOF* gene mutation that were responsible for causing ANSD, and 6 cases of mutations in *TBC1D24, LARS2, TIMM8A, MITF*, and *WFS1* genes. Our research related to the etiological analysis of ANSD expanded the *OTOF* gene mutation spectrum and indicated the pathogenic role of *TBC1D24, LARS2, TIMM8A, MITF*, and *WFS1* genes in ANSD.

## Methods

### Subjects and clinical evaluations

Among 741 hearing-impaired patients undergoing genetic counseling, a total of 9 families were recruited in this study, from the department of otolaryngology-head and neck surgery of Xin Hua Hospital affiliated with Shanghai Jiao Tong University School of Medicine. Approvals were achieved by all individuals and their family members with informed consent prior to the study. Questionnaires were designed to collect the subjects' medical histories. Otological examinations were then conducted to evaluate the auditory conditions of the subjects, which included otoscopy, auditory brainstem response (ABR), pure-tone audiometric examination (PTA), cochlear microphonic potential (CM), and distortion product otoacoustic emission (DPOAE). Finally, we made the diagnosis of ANSD according to the abnormal results of ABR tests and the normal results of DPOAE/CM. This research was approved by the Ethics Committee of Xin Hua Hospital affiliated with Shanghai Jiao Tong University School of Medicine (No. XHEC-D-2021-047).

### Next-generation sequencing

The genomic DNA of family members was extracted from peripheral blood leukocytes. The panel of 140 deafness-causative genes of the proband in family A diagnosed with ANSD was captured and sequenced by the Illumina sequencing platform and Hiseq X sequencer (Illumina, San Diego, CA, United States). The panel of 415 deafness-causative genes of 8 other probands diagnosed with ANSD was captured and sequenced by the Illumina sequencing platform and NextSeq 500 sequencer (Supplementary Table 1). Then, non-sense variants, frameshift, and splicing site were taken into further consideration with allele frequencies below 0.0005 for dominant inheritance and 0.005 for recessive inheritance in the 1,000 Genomes Project. Moreover, Mutation taster and SIFT software were then applied to evaluate the possible pathogenicity (20). Through targeted next-generation sequencing, potential causative variants could be detected, which were then confirmed by Sanger sequencing in each individual. Meanwhile, co-segregation analysis was also conducted for all family members if available. The three-dimensional structure of the mutation protein was built individually by SWISS-MODEL (https://swissmodel.expasy.org/) or visualized individually by Swiss-PdbViewer (http://spdbv.vital-it.ch/).

### Mini-gene assay

Mini-gene assay was used to study whether the mutation near the splicing site of c.2406 + 2insT in *OTOF* affects the formation of mRNA by using vectors constructed *in vitro*. First of all, wild-type and mutant gene inserts were amplified from the genomic DNA of the proband and her mother in family B by nest PCR. Peripheral primers from forward to reverse and 5′- to 3′-, the same as below, of nest PCR were as follows: GTTGAAGTTCCCTGAAGCTCAGCCAGCTC and CCCTGGTCAGAGCTGCCCTG. Inner primers of nest PCR were as follows: CTCACTCCCCTGATCAACAG and GAAGAGCGTCTTGACCTTGGC. The inserts were cloned into the pcDNA3.1 and pEGFP-C1 vectors with multiple cloning sites. The mini-gene constructs were then transfected into HeLa and 293T cells separately using Lipofectamine^TM^ 3,000 transfection reagent (Thermo Fisher Scientific, Waltham, USA). Cells were harvested 48 h after transfection. In the end, the total RNA was extracted by Trizol (TaKaRa, Kyoto, Japan), reverse transcribed into cDNA by Hifair^TM^ reverse transcriptase (YEASEN, Shanghai, China), and the amplified products were analyzed by electrophoresis of agarose and Sanger sequencing, to confirm whether the mutant vector is spliced as wild type. The primers used to amplify the inserts cloned into pcDNA3.1 were as follows: AAACTTAAGCTTATGTGCCGCTTCCTCTCCCTCGCTG and TAGTGGATCCCTCGTCCGCCAGGAAGCGCA. The primers used to amplify the inserts cloned into pEGFP-C1 were as follows: GCTCAAGCTTCCTGCCGCTTCCTCTCCCTCGCTG and CCGCGGTACCCTCGTCCGCCAGGAAGCGCA.

## Results

### Clinical evaluations

Probands in the 9 Chinese families, including 5 girls and 4 boys, aged from 15 to 53 months were diagnosed with ANSD according to the abnormal results of ABR tests and normal results of DPOAE/CM. In addition to the audiological diagnosis of the probands, other members of the families need to be specifically described as follows. The father of the family G had normal hearing but excessive freckles all over his body. The father of the family H showed asymmetrical hearing loss with severe hearing loss in the right ear and mild hearing loss in the left ear in PTA testing. The proband of the family I was stunted and could not walk independently at 28 months old. His father showed normal hearing without other physical abnormalities. The parents of other families showed normal hearing without other physical abnormalities.

### Genetic findings

**Figure 1:**
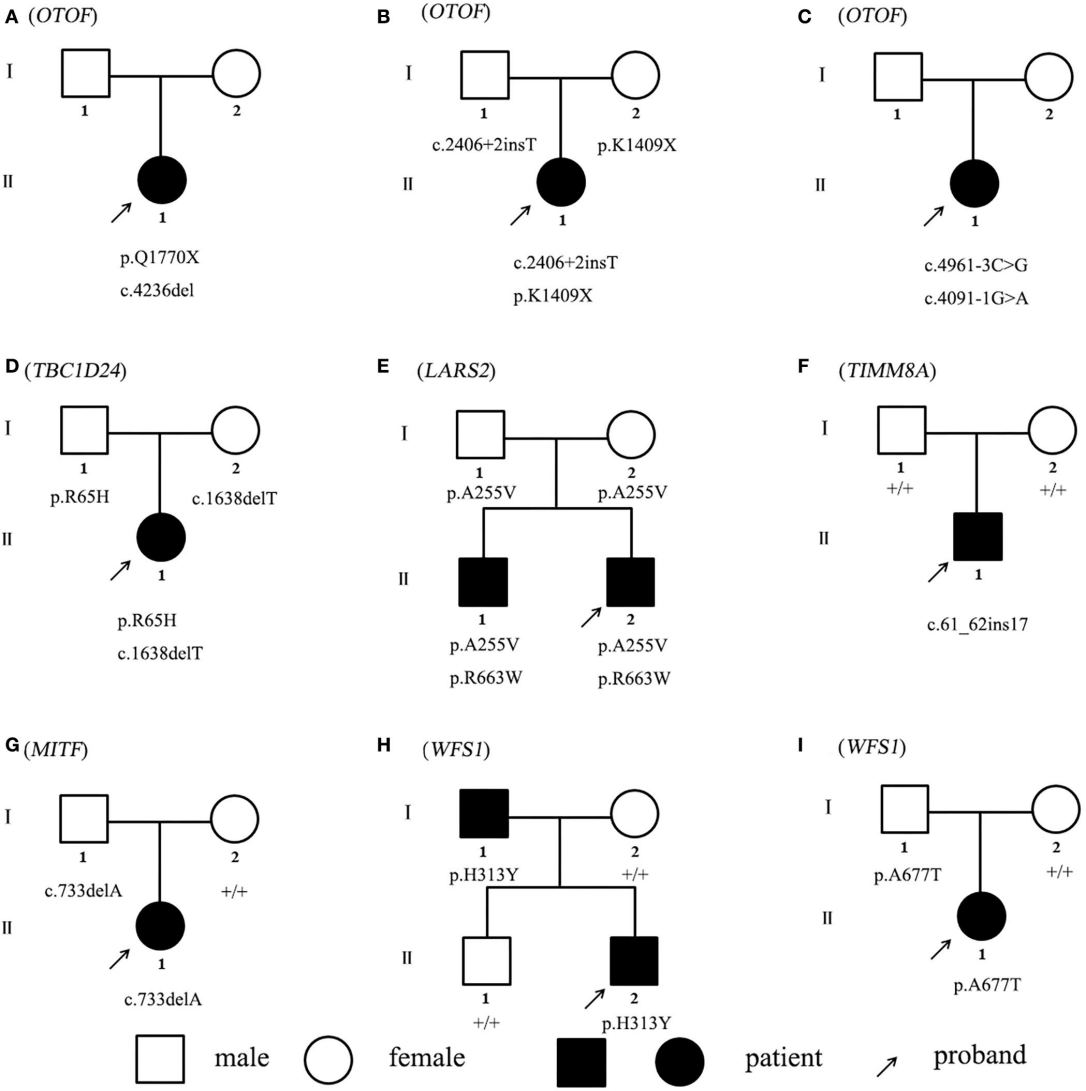
**(A–I)** Pedigrees of nine families. + represents the wild type after Sanger sequencing.

Non-sense, frameshift, and splicing site variants with allele frequencies below 0.0005 for dominant inheritance and 0.005 for recessive inheritance were screened to detect potential causative variants by targeted NGS. Candidate causative variants are shown in Table 1. In the five recessive families, bi-allelic mutations identified in known deafness genes were confirmed by parental genotyping, including p.Q1770X + c.4263delC in *OTOF* (OMIM 603681) for Family A, c.2406 + 2insT + p.K1409X in *OTOF* (OMIM 603681) for Family B, c.4961-3C > G + c.4091-1G > A in *OTOF* (OMIM 603681) for Family C, p.R65H + c.1638delT in *TBC1D24* (OMIM 613577) for Family D, and p.A255V + p.R663W in *LARS2* (OMIM 604544) for Family E (Table 1). While in the four dominant families, we identified four dominant deafness-related heterozygous variants, c.61_62insGGACCCGCAGTTGCAGC in *TIMM8A* (OMIM 300356) for Family F, c.733delA in *MITF* (OMIM 156845) for Family G, p.H313Y in *WFS1* (OMIM 606201) for Family H, and p.A677T in *WFS1* (OMIM 606201) for Family I, co-segregating with the hearing impairment (Figure 1). The co-segregation of the reported mutations was confirmed with the hearing phenotype in these family members by Sanger sequencing (Supplementary Table 2). Among them, there are 9 novel mutations that have not been reported previously in this study. According to ACMG guidelines, most of these mutations were classified as likely pathogenic, although there are three variations of uncertain significance, we suggest that they are important to study the etiology of the family, thus we have included them in the table (Table 1, Supplementary Figure).

**Table 1:** The gene mutations of the 9 families.

**Gene**	**Mutation type**	**Nucleotide change (transcript version)**	**Amino acid change**	**InterAcmg**	**Mutationtaster**	**Pathogenic grade**	**SIFT (score)**	**Allele frequency in controls**	**Reference**
**Autosomal recessive**									
*OTOF*	Stop coden	c.5308C > T (NM_194248)	p.Gln1770^*^	PVS1, PM2	Disease_causing_automatic (1)	Likely pathogenic	–	0/1,000	Novel
	Frameshift	c.4236del (NM_194248)	p.Glu1414Serfs*108	PVS1, PM2	–	Likely pathogenic	–	0/1,000	Novel
*OTOF*	Stop coden	c.4225A > T (NM_194248)	p.K1409^*^	PVS1, PM3_Strong, PM2	Disease_causing_automatic (1)	Pathogenic	–	0/1,000	PMID:23767834
	Splicing	c.2406 + 2_2406 + 3insT (NM_194248)	–	PVS1, PM2	–	Likely pathogenic	–	0/1,000	Novel
*OTOF*	Splicing	c.4961-3C > G (NM_194248)	–	PM3_Strong, PM2	–	Likely pathogenic	–	0/1,000	PMID:23767834
	Splicing	c.4091-1G > A (NM_194248)	–	PVS1,PM2	Disease_causing (1)	Likely pathogenic	–	0/1,000	Novel
*TBC1D24*	Missense	c.194G > A (NM_001199107)	p.R65H	PM2,PM5	Disease_causing (1)	Uncertain	Probably_damaging (0.997)	0/1,000	Novel
	Frameshift	c.1638delT (NM_001199107)	p.A547Pfs*21	PVS1_PM4,PM2	–	Uncertain	–	0/1,000	Novel
*LARS2*	Missense	c.764C > T (NM_015340)	p.A255V	PM2	Polymorphism (0.711)	Uncertain	Tolerated (0.119)	0/1,000	Novel
	Missense	c.1987C > T (NM_015340)	p.R663W	PM3_Strong,PM2,PP3	Disease_causing (1)	Likely pathogenic	Damaging (0)	0/1,000	PMID:28708303
**Autosomal dominant**									
*TIMM8A*	Frameshift	c.61_62insGGACCCGCAGT TGCAGC (NM_004085)	p.H21Rfs*11	PVS1,PM2	–	Likely pathogenic	–	0/1,000	Novel
*MITF*	Frameshift	c.733delA (NM_000248)	p.T245Pfs*3	PVS1,PM2	–	Likely pathogenic	–	0/1,000	Novel
*WFS1*	Missense	c.937C > T (NM_006005)	p.H313Y	PM3_Strong,PM2,PP3	Disease_causing (1)	Likely pathogenic	Tolerated (0.082)	0/1,000	PMID:16151413
*WFS1*	Missense	c.2029G > A (NM_006005)	p.A677T	PM3_Strong,PM1,PM2,PP3	Disease_causing (0.999)	Likely pathogenic	Tolerated (0.149)	0/1,000	PMID:26969326

* symbol indicates the stop codon; - symbol indicates no relevant information.

### Mini-gene assay of the splicing site

**Figure 2:**
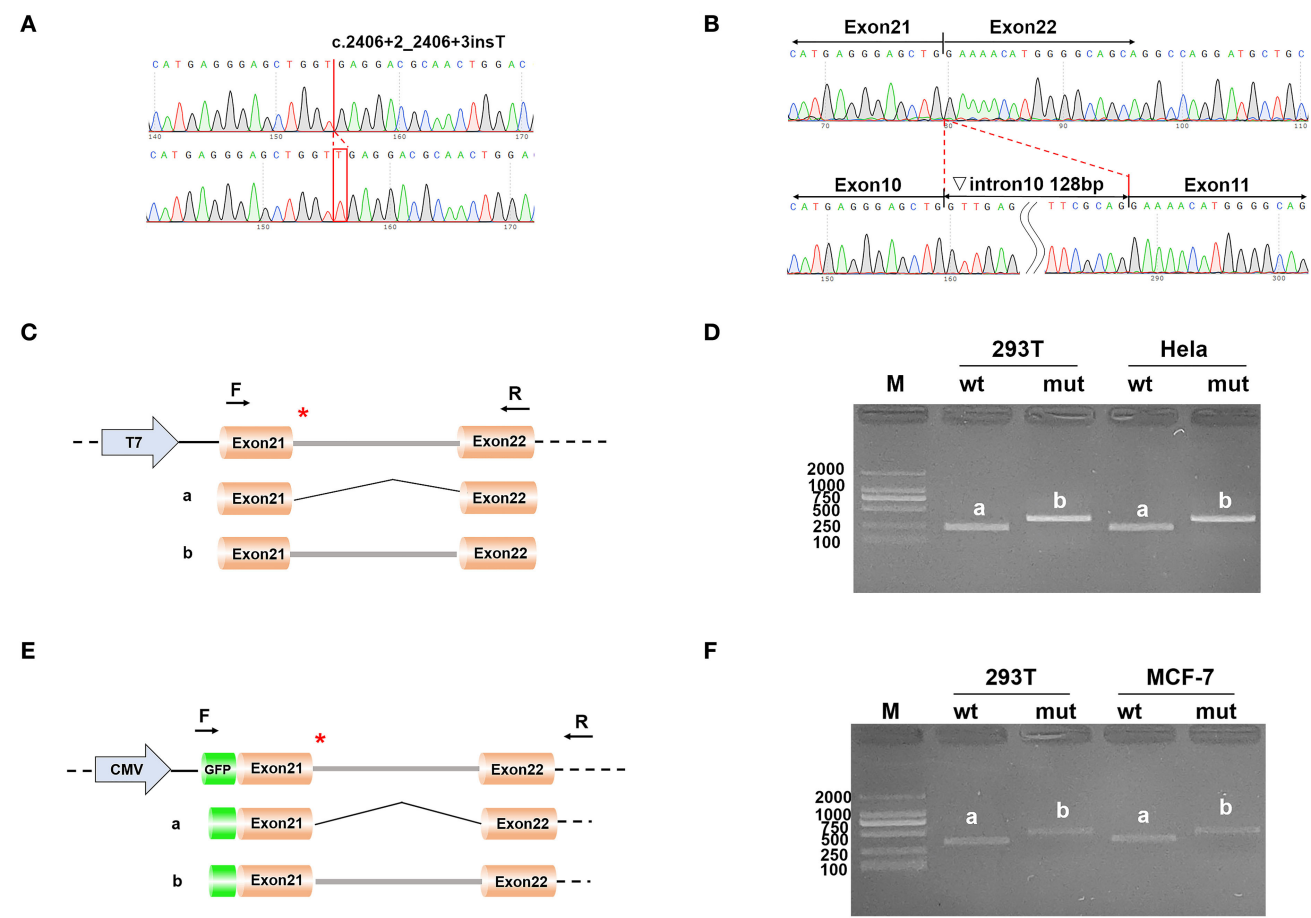
**(A)** The result of the selected clones by sequencing; **(B)** The result of final expressed products of cells by sequencing; **(C)** Construction strategy of pcDNA3.1 vectors; **(D)** Electrophoretic results of expression products of pcDNA3.1 vectors in cell lines; **(E)** Construction strategy of pEGFP-C1 vectors; and **(F)** Electrophoretic results of expression products of pEGFP-C1 vectors in cell lines.

Two different strategies for constructing mini-genes were used (Figure 2). The wild type and mutant mini-genes were inserted into pcDNA3.1 and pEGFP-C1 vectors. A total of four recombinant vectors were transfected into different cell lines of 293T, Hela, and MCF-7. A total of eight samples were collected 48 h after transfection. The mini-gene construction strategy of pcDNA3.1-otof-wt/mut is to insert the complete fragment of exon21 (91 bp)—intron21 (127 bp)—exon22 (117 bp) of *OTOF* gene into pcDNA3.1 vector and observe whether there is abnormal splicing between exon21 and exon22 in c.2406 + 2 ins T *OTOF* mutant after transfection.

The results of RT-PCR indicated that the wild type had an electrophoretic band of the expected size (241 bp) in 293T and Hela cells, and the mutant was slightly larger than the wild type. Both wild type and mutant bands were sequenced. The sequencing results showed that the wild type was normal and its splicing mode was exon21–exon22; the mutant retained all intron21 of 128 bp, that is, the splicing mode of the mutant was exon21–intron21–exon22. We obtained the same results on the pEGFP-C1 vector, with 359 bp in the wild type and a larger electrophoretic band in the mutant (Figure 2).

## Discussion

Auditory neuropathy spectrum disease is caused by defects in genes, which have been proven in previous studies in the recent two decades (3). Mutations in *OTOF* (OMIM 603681), which was the first identified gene of congenital ANSD, were the most common cause of these genetic defects (21). In previous studies, it has been elucidated that the *OTOF* gene located on chromosome 2p23.1 consists of 48 exons and encodes otoferlin, which is located in the basolateral region of the adult mammalian cochlea, and is mainly expressed in the inner hair cells, participates in the connection activities in afferent synapses. A reduction of synaptic vesicle exocytosis was observed at ribbon synapses with mutations of *OTOF* (22). Therefore, the auditory nerve function of patients with bi-allelic *OTOF* mutations could be assumed to be intact, and the site of the lesion is presumed to be presynaptic in the auditory neuron. Theoretically, good cochlear implant performance could be anticipated in patients with ANSD with *OTOF* gene mutations. This study expanded the mutation spectrum of the *OTOF* gene with four novel mutations of the *OTOF* gene identified.

The disorders associated with *TBC1D24* are characterized by some features which were described as distinct, recognized phenotypes originally, including deafness, epilepsy, intellectual disability, and osteodystrophy. The diagnosis of a *TBC1D24*-associated disorder is confirmed in an individual with bi-allelic *TBC1D24* pathogenic mutations, and the pattern of inheritance of *TBC1D24* mutation is autosomal recessive (23). *TBC1D24* is assumed to be a suitable candidate gene for ANSD for its involvement in the central nervous system and expression in the spiral ganglion (16). In this study, the family members denied that the patient had any symptoms other than deafness. Verification of future research is needed due to the two mutations of this study were classified as uncertain according to ACMG guidelines. Until now, our study is the first report of ANSD caused by *TBC1D24* mutations.

*LARS2* variants are associated with disorders called Perrault syndrome (OMIM 615300) in most studies, characterized by premature ovarian failure and sensorineural hearing deafness (24). More recently, bi-allelic *LARS2* variants have been reported to lead to Perrault syndrome with neurological symptoms (25). In this study, the patients also showed symptoms of ANSD. In our study, the proband and his siblings were young boys and only showed hearing problems.

*TIMM8A* located in Xq22 encodes a small protein located in the mitochondrial intermembrane space associated with Deafness-dystonia-optic neuronopathy (DDON syndrome) also called Mohr–Tranebjaerg syndrome (MTS) (26). This was the first case of an 11-year-old Dutch boy with dystonia and deafness to report a *TIMM8A* mutation (27). Three patients with MTS with primary auditory neuropathy in China were the first to report *TIMM8A* variations (28). In this study, the patient developed auditory neuropathy symptoms, and the mutation was novel and *de novo*.

Heterozygous mutations in the *MITF* gene are strongly related to pigmentation disorders and deafness called Waardenburg Syndrome 2A (WS2A). Compound heterozygotes were recently elucidated in a novel syndrome involving coloboma, osteopetrosis, microphthalmia, macrocephaly, albinism, and deafness. Our previous studies have revealed that WS2A caused by *MITF* mutations is clinically related to excess freckles in Han Chinese deaf patients (29). To our knowledge, this is the first study reporting WS2A as a primary symptom of an ANSD.

*WFS1* mutations lead to type 6/14/38 autosomal dominant non-syndromic deafness (DFNA) and Wolfram syndrome 1, an autosomal recessive neurodegenerative disease including deafness, optic nerve atrophy, and diabetes insipidus (30). In this study, the inheritance pattern of Family H was autosomal dominant, while the proband exhibited symptoms of ANSD and the father exhibited symptoms of Wolfram syndrome 1 including deafness and optic nerve atrophy. The inheritance pattern of Family I was incomplete autosomal dominant, with the mutant father having no phenotype, while the mutant daughter exhibits an auditory neuropathy phenotype and developmental delay.

## Conclusion

Our results from limited samples suggest that *OTOF* plays a leading role and *WFS1* plays a secondary role in the genetic etiology analysis of ANSD, which together constitute a complex genetic etiology of ANSD. Our results extend the mutation spectrum of the *OTOF* gene and indicate that the genetic etiology of ANSD may be related to gene mutations of *TBC1D24, LARS2, TIMM8A, MITF*, and *WFS1*.

## Data availability statement

The datasets presented in this study can be found in online repositories. The name of the repository and accession number can be found below: National Center for Biotechnology Information (NCBI) BioProject, https://www.ncbi.nlm.nih.gov/bioproject/, PRJNA861021.

## Ethics statement

The studies involving human participants were reviewed and approved by Ethics Committee of Xin Hua Hospital affiliated to Shanghai Jiao Tong University School of Medicine. Written informed consent to participate in this study was provided by the participants' legal guardian/next of kin.

## Author contributions

LS wrote the article. ZL, JZ, JS, and XW collected the data. JY designed this study. All authors contributed to the article and approved the submitted version.
