# Supplementary material for: Genetic etiological analysis of auditory neuropathy spectrum disorder by next-generation sequencing
Source: Front Neurol. 2022 Dec 8;13:1026695. doi: 10.3389/fneur.2022.1026695 (PMC9772003; doi:10.3389/fneur.2022.1026695)
Supplement: Supplementary file 1 [file Data_Sheet_1.docx]

Supplementary Figure

**A**

*OTOF*; chr2:26690093; NM_194248.2:c.4236del(p.Glu1414Serfs*108)

There is no 3D structure in SWISS MODEL.

*OTOF*; chr2:26684789; NM_194248.2:c.5308C>T(p.Gln1770*)

There is no 3D structure in SWISS MODEL.

B

*OTOF*; chr2:26690235; NM_194248.2:c.4225A>T(p.K1409*)

There is no 3D structure in SWISS MODEL.


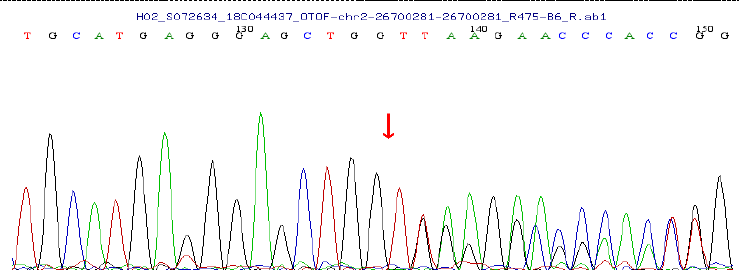


*OTOF*; chr2:26700281-26700281; NM_194248.2:c.2406+2_2406+3insT

There is no 3D structure in SWISS MODEL.

**C**


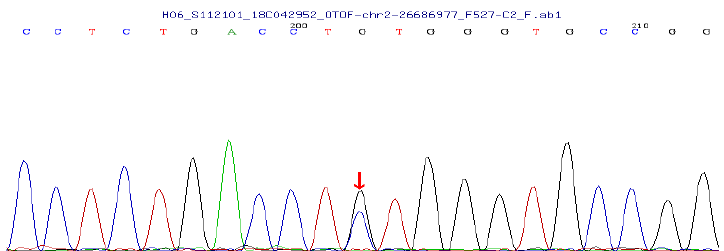


*OTOF*; chr2:26686977; NM_194248.2:c.4961-3C>G

There is no 3D structure in SWISS MODEL.


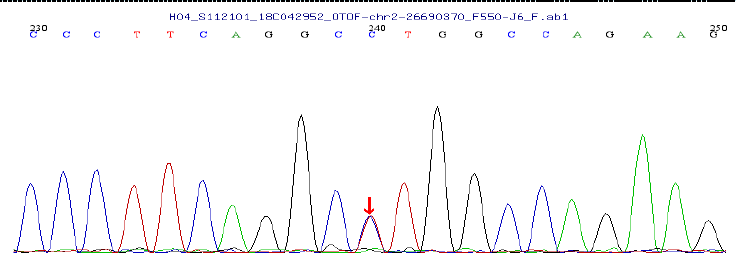


*OTOF*; chr2:26690370; NM_194248.2:c.4091-1G>A

There is no 3D structure in SWISS MODEL.

**D**


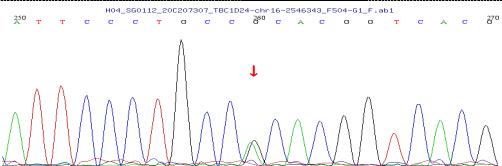


*TBC1D24*; chr16:2546343; NM_001199107:c.194G>A (p.R65H)

a
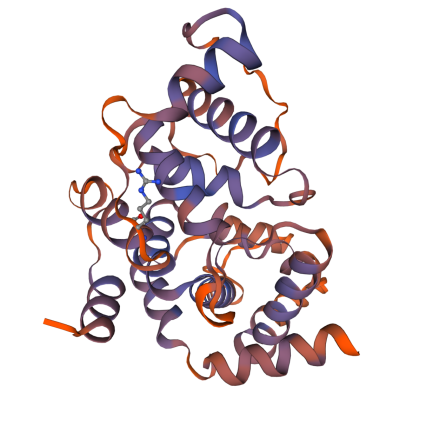
 b
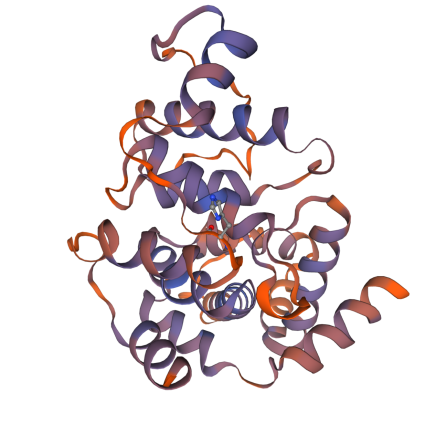


a is the 3D structure of Arg in the 65 site, b is the 3D structure of His in the 65 site.


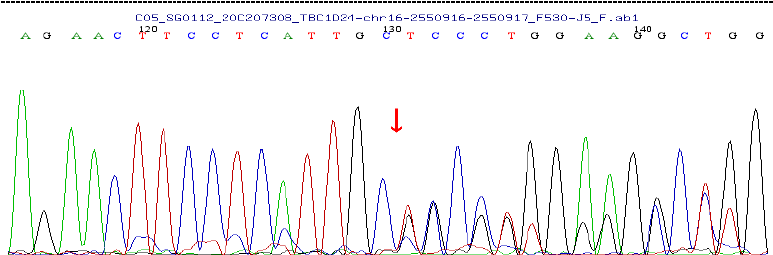


*TBC1D24*; chr16:2550916-2550917; NM_001199107:c.1638delT (p.A547Pfs*21)

There is no 3D structure in SWISS MODEL.

**E**


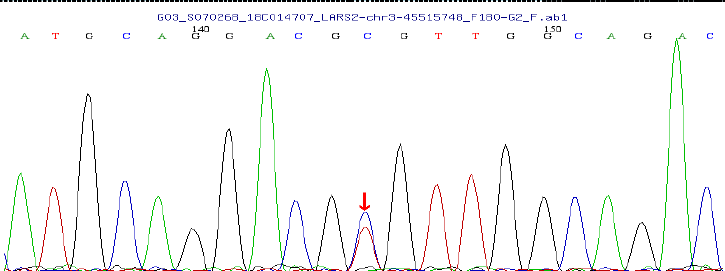


*LARS2*; chr3:45515748; NM_015340:c.764C>T (p.A255V)

a
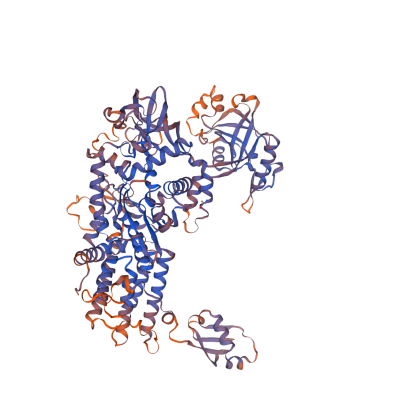
 b
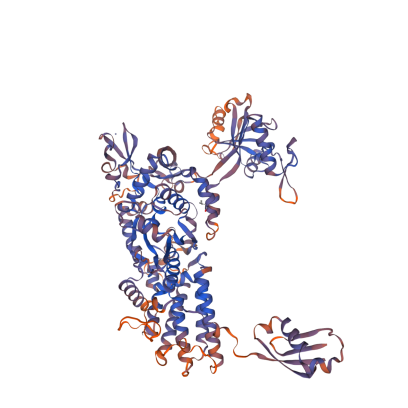


a is the 3D structure of Ala in the 225 site, b is the 3D structure of Val in the 225 site.


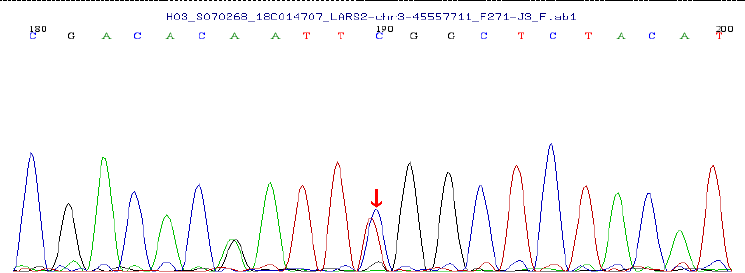


*LARS2*; chr3:45557711; NM_015340:c.1987C>T (p.R663W)

a
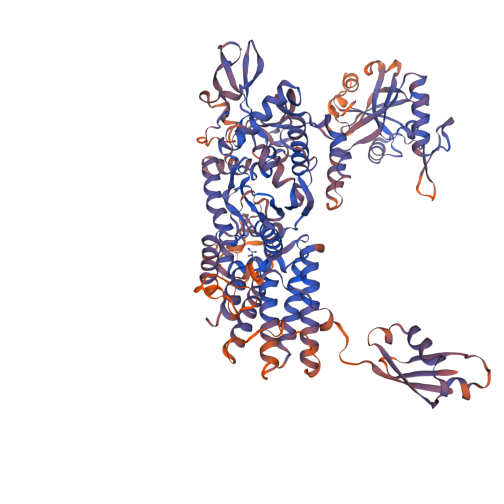
 b
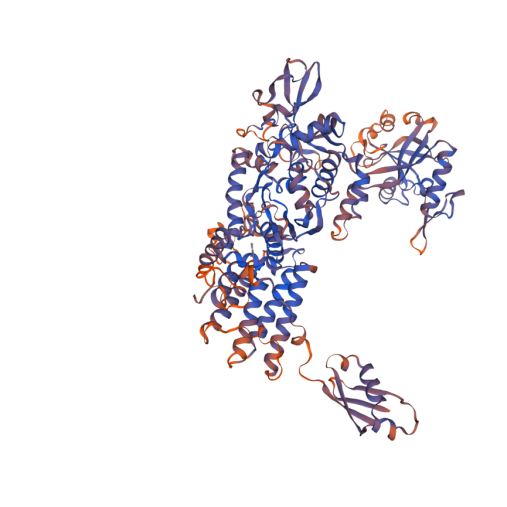


a is the 3D structure of Arg in the 663 site, b is the 3D structure of Trp in the 663 site.

**F**


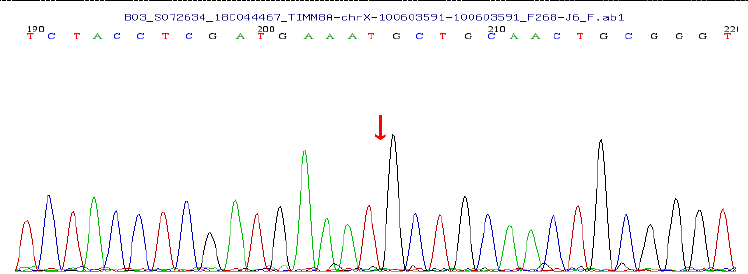


*TIMM8A*; chrX: 100603591-100603591; NM_004085: c.61_62insGGACCCGCAGTTGCAGC (p.H21Rfs*11)

There is no 3D structure in SWISS MODEL.

**G**


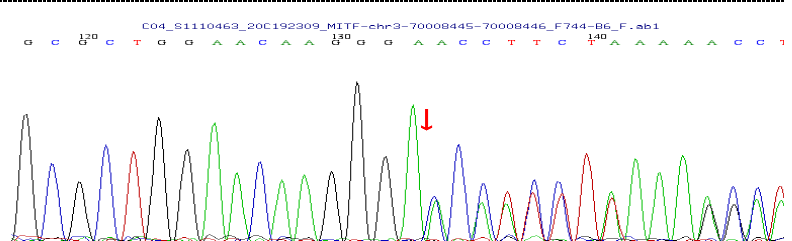


*MITF*; chr3:70008445-70008446; NM_000248: c.733delA (p.T245Pfs*3)

There is no 3D structure in SWISS MODEL.

H


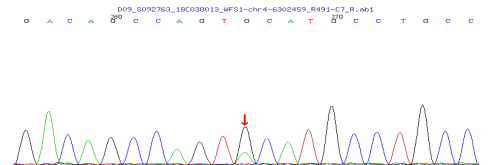


*WFS1*; chr4: 6302459; NM_006005: c.937C>T (p.H313Y)

There is no 3D structure in SWISS MODEL.

**I**


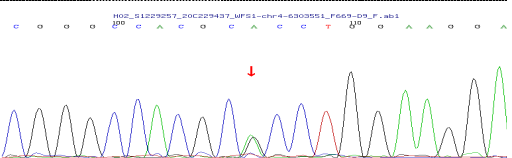


*WFS1*; chr4:6303551; NM_006005: c.2029G>A (p.A677T)

There is no 3D structure in SWISS MODEL.
