# Supplementary figures and images for: Genetic etiological analysis of auditory neuropathy spectrum disorder by next-generation sequencing
Source: Front Neurol. 2022 Dec 8;13:1026695. doi: 10.3389/fneur.2022.1026695 (PMC9772003; doi:10.3389/fneur.2022.1026695)

**Supplementary Table S1.** 415 deafness genes targeted for the next-generation sequencing.


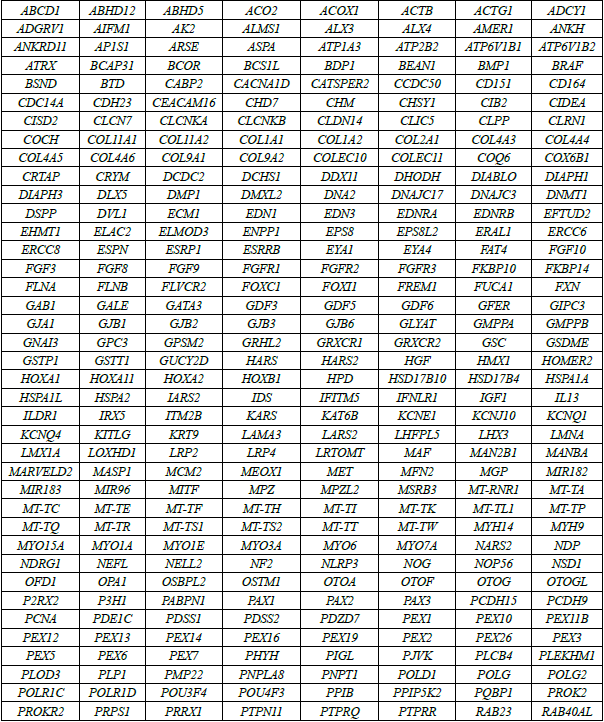

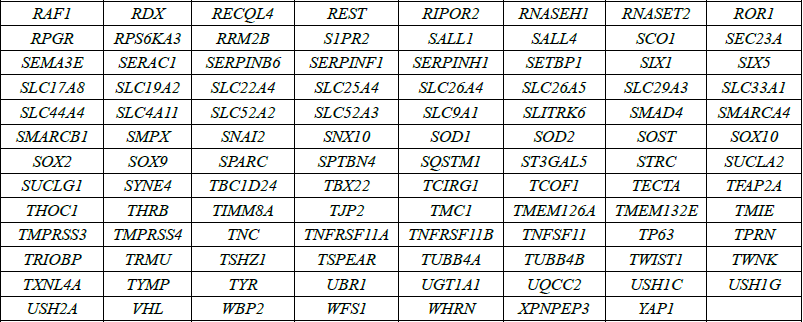

Supplement: Supplementary file 2 [file Table_1.docx]
