# Supplementary material for: Genetic etiological analysis of auditory neuropathy spectrum disorder by next-generation sequencing
Source: Front Neurol. 2022 Dec 8;13:1026695. doi: 10.3389/fneur.2022.1026695 (PMC9772003; doi:10.3389/fneur.2022.1026695)
Supplement: Supplementary file 3 [file Table_2.docx]

**Supplementary table S2.** Excluded gene mutation sites

| **Family number** | **Gene** | **Nucleotide change (transcript version)** | **Amino acid change** | **Variant form** | **Mode of inheritance** | **Variation classification** |
| --- | --- | --- | --- | --- | --- | --- |
| AN-1 | CDH23 | c.3487C>G(NM_022124.5) | p.Pro1163Ala | Het | AR/DR | unknown |
|  | GIPC3 | c.679G>T(NM_133261.2) | p.Gly227Trp | Het | AR | unknown |
|  | COL4A3 | c.3476G>A(NM_000091.4) | p.Arg1159His | Het | AD/AR | unknown |
|  | CACNA1D | c.2466+5G>A(NM_000720.3) | - | Het | AR | unknown |
|  | PCDH15 | c.2884C>T(NM_033056.3) | p.Arg962Cys | Het | AR/DR | unknown |
| AN-2 | TECTA | c.5832T>A（NM_005422） | p.H1944Q | Het | AD/AR | unknown |
|  | LOXHD1 | c.2359C>T(NM_144612) | p.R787C | Het | AR | unknown |
|  | OTOG | c.1325C>T(NM_033056) | p.A442V | Het | AR | unknown |
|  | PCDH15 | c.552T>A(NM_033056) | p.N184K | Het | AR | unknown |
|  | PDZD7 | c.490C>T(NM_001195) | p.R164W | Het | AR | unknown |
|  | USH2A | c.13478G>A(NM_206933) | p.R4493H | Het | AR | unknown |
|  | TYR | c.896G>A(NM_000372) | p.R299H | Het | AR | unknown |
| AN-3 | OTOF | c.145C>T(NM_194248) | p.R49W | Het | AR | unknown |
|  | PTPRQ | c.3170C>T(NM_001145026) | p.P1057L | Het | AD/AR | unknown |
|  | SLC26A4 | c.563T>C(NM_000441) | p.I188T | Het | AR | unknown |
|  | PCDH15 | c.4812G>T(NM_033056) | p.R1604S | Het | AR/DR | unknown |
|  | TSPEAR | c.421T>C(NM_144991) | p.W141R | Het | AR | unknown |
| AN-4 | SLC26A4 | c.919-2A>G(NM_000441) | - | Het | AR | pathogic |
|  | NLRP3 | c.226G>A(NM_004895) | p.A76T | Het | AD | unknown |
|  | PEX1 | c.2200G>A(NM_000466) | p.V734I | Het | AR | unknown |
|  | FAT4 | c.5443C>T(NM_024582) | p.R1815C | Het | AR | unknown |
|  | OSTM1 | c.392G>A(NM_014028) | p.R131Q | Het | AR | unknown |
| AN-5 | CCDC50 | c.1230C>A(NM_178335) | p.D410E | Het | AD | unknown |
|  | TNC | c.4498C>G(NM_002160) | p.P1500A | Het | AD | unknown |
|  | TNC | c.2627G>T(NM_002160) | - | Het | AD | unknown |
| AN-6 | MYO15A | c.3742C>T(NM_016239) | p.R1248W | Het | AR | unknown |
|  | MYO15A | c.9478C>T(NM_016239) | p.L3160F | Het | AR | unknown |
|  | OTOF | c.2180A>G（NM_194248） | p.N727S | Het | AR | unknown |
|  | WHRN | c.1649C>G(NM_015404) | p.T550S | Het | AR | unknown |
|  | ADGRV1 | c.5576A>G(NM_032119) | p.H1859R | Het | AR | unknown |
| AN-7 | ERCC6 | c.2962A>G(NM_000124) | p.K988E | Het | AR | unknown |
|  | AMER1 | c.3145C>T(NM_152424) | p.R1049X | Het | XLD | unknown |
|  | TRIOBP | c.3050G>A(NM_001039141) | p.C1017Y | Het | AR | unknown |
|  | TRIOBP | c.965C>T(NM_001039141) | p.A322Y | Het | AR | unknown |
|  | MYO1E | c.1840G>C(NM_004998) | p.E614Q | Het | AR | unknown |
|  | PAX1 | c.101G>C(NM_006192) | p.R34P | Het | AR | unknown |
|  | OTOF | c.1198A>G(NM_194248) | p.I400V | Het | AR | unknown |
|  | SIPR2 | c.919A>T(NM_004230) | p.R307W | Het | AR | unknown |
| AN-8 | WFS1 | c.2596G>A(NM_006005) | p.D866N | Het | AR/AD | unknown |
|  | CLRN1 | c.472+5T>C(NM_001195794) | - | Het | AR | unknown |
|  | GJB2 | c.109G>A(NM_004004) | p.V37I | Het | AR | unknown |
|  | KARS | c.685T>C(NM_001130089) | p.Y229H | Het | AR | unknown |
|  | LAMA3 | c.6730G>T(NM_198129) | p.A2244S | Het | AR | unknown |
|  | TRIOBP | c.4484A>T(NM_001039141) | p.E1495V | Het | AR | unknown |
| AN-9 | COL9A1 | c.995G>T(NM_001851) | p.G332V | Het | AR/AD | unknown |
|  | CDH23 | c.754-7T>G(NM_022124) | - | Het | AR/AD | unknown |
|  | CDH23 | c.5389G>C（NM_022124） | p.V1797L | Het | AR/AD | unknown |
|  | SALL4 | c.2449A>G（NM_020436） | p.T817A | Het | AD | unknown |
|  | DCHS1 | c.7465G>A(NM_003737) | p.V2489M | Het | AR/AD | unknown |
|  | DCHS1 | c.9766C>T(NM_003737) | p.P3256S | Het | AR/AD | unknown |
|  | LAMA3 | c.3049A>G(NM_000227) | p.I1017V | Het | AR | unknown |
|  | DNAJC3 | c.1323T>A(NM_006260) | p.D441E | Het | AR | unknown |
|  | ABHD12 | c.802G>T(NM_001042472) | p.A268S | Het | AR | unknown |
|  | RPS6KA3 | c.1353+5A>T(NM_004586) | - | Het | XLD | unknown |

-, No information
